# Effects of Task on Reading Performance Estimates

**DOI:** 10.3389/fpsyg.2020.02005

**Published:** 2020-08-07

**Authors:** Tiffany Arango, Deyue Yu, Zhong-Lin Lu, Peter J. Bex

**Affiliations:** ^1^Department of Psychology, Northeastern University, Boston, MA, United States; ^2^College of Optometry, The Ohio State University, Columbus, OH, United States; ^3^Division of Arts and Sciences, NYU Shanghai, Shanghai, China; ^4^Center for Neural Science, Department of Psychology, New York University, New York, NY, United States; ^5^NYU-ECNU Institute of Brain and Cognitive Science at NYU Shanghai, Shanghai, China

**Keywords:** reading, reading acuity, critical print size, reading speed, MNREAD

## Abstract

Reading is a primary problem for low vision patients and a common functional endpoint for eye disease. However, there is limited agreement on reading assessment methods for clinical outcomes. Many clinical reading tests lack standardized materials for repeated testing and cannot be self-administered, which limit their use for vision rehabilitation monitoring and remote assessment. We compared three different reading assessment methods to address these limitations. Normally sighted participants (*N* = 12) completed MNREAD, and two forced-choice reading tests at multiple font sizes in counterbalanced order. In a word identification task, participants indicated whether 5-letter pentagrams, syntactically matched to English, were words or non-words. In a true/false reading task, participants indicated whether four-word sentences presented in RSVP were logically true or false. The reading speed vs. print size data from each experiment were fit by an exponential function with parameters for reading acuity, critical print size and maximum reading speed. In all cases, reading speed increased quickly as an exponential function of text size. Reading speed and critical print size significantly differed across tasks, but not reading acuity. Reading speeds were faster for word/non-word and true/false reading tasks, consistent with the elimination of eye movement load in RSVP but required larger text sizes to achieve those faster reading speeds. These different reading tasks quantify distinct aspects of reading behavior and the preferred assessment method may depend on the goal of intervention. Reading performance is an important clinical endpoint and a key quality of life indicator, however, differences across methods complicate direct comparisons across studies.

## Introduction

Reading is a complex visuo-cognitive process that can be negatively affected by vision loss. Reading difficulty is a primary complaint for low vision patients, and improving reading ability is a major component of vision rehabilitation (Elliott et al., 1997; Stelmack et al., 2017). Reading performance is a key vision-related quality of life indicator, with lower scores on quality of life (MacDQoL questionnaire) being associated with visual impairment severity (Mitchell et al., 2008). Decreased visual function is also associated with increased depressive symptoms, and significantly impairs performance on activities of daily living including mobility tasks, preparing meals and taking prescription medicines (Binns et al., 2012). Reading behavior encapsulates multiple aspects of visual, phonological and semantic processing and requires efficient integration of these processes. Vision loss can impair reading performance at one or more of these processes.

Single letter visual acuity is also associated with quality of life in low vision patients, although it may not be the best predictor of visual function. Visual acuity is often measured with a Snellen eye chart that uses high-contrast black letters on a white background (Ferris and Bailey, 1996). Despite being a common functional endpoint, visual acuity might remain unchanged during early stages of diseases such as glaucoma and maculopathies (Sunness et al., 1997), as well as insensitive to subtle changes in visual performance (Ahn et al., 1995). Reading performance may be a better functional endpoint, as it is sensitive to both oculomotor characteristics, such as fixation stability (Crossland et al., 2004) and to physical characteristics of text, such as font style, size and spacing (Legge et al., 1989; Chung et al., 1998).

Reading difficulty that is attributed to factors like poor resolution acuity can often be alleviated with magnification alone. However, in many low vision diseases, reading difficulty is compounded by various spatiotemporal and oculomotor factors not easily remedied with magnification (Dickinson and Fotinakis, 2000) and each factor may account for a unique contribution to reading impairment. This is further complicated by individual differences in visual ability among low vision populations. Some patients may undergo mild to moderate acuity loss, but poor fixation stability, while others may experience significant acuity loss, but exhibit better oculomotor control during reading (Sunness et al., 1996). Additionally, some patients with central vision loss may adopt multiple preferred retinal locations (PRLs) depending on the task, and in fact visual acuity may not be the best at the selected PRL (Bernard and Chung, 2018). These individual differences make it difficult to select a reading task that is both sensitive and specific for rehabilitation training.

**FIGURE 1:**
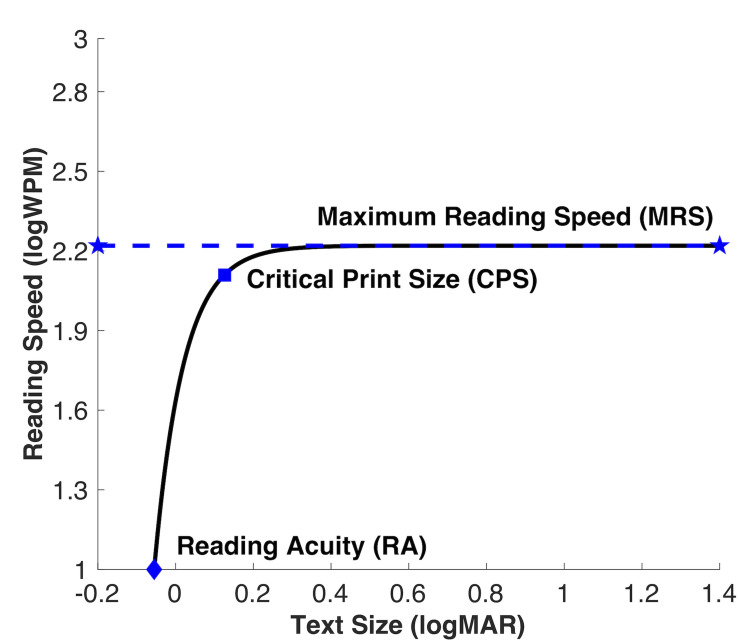
Reading curve is defined by three parameters: asymptotic duration level corresponding to maximum reading speed, critical print size (smallest text size achieved at maximum reading speed), and reading acuity (smallest text size that can be resolved).

Clinicians and ophthalmic researchers often quantify reading performance by measuring the relationship between reading speed and print size (Mansfield et al., 1996; O’Brien et al., 2005; Bernard et al., 2013), commonly referred to as a reading function (Figure 1). Reading speed as a function of text size can be fit with an exponential-decay function, and the canonical shape reflects a steep rise at smaller text sizes and horizontal asymptote duration at larger print sizes (Cheung et al., 2008). The asymptote of the fitted function represents the maximum reading speed (MRS). Critical print size (CPS) is the smallest text size achieved at MRS, and can be estimated as the text size on the fitted function that yields a criterion percentage of the MRS, typically 75–95%. A higher criterion indicates a more conservative estimated CPS. Reading acuity (RA) is computed as the smallest text size that can be resolved. This reading relationship holds for normally sighted and low vision individuals.

Standardized reading charts that measure reading speed as a function of print size include: MNREAD Charts, SKread Chart, Bailey-Lovie Charts, RADNER Reading Charts, the Colenbrander English Continuous Text Near Vision Cards and Pepper Visual Skills for Reading (VSRT). Letter size in these charts progress logarithmically, and researchers may examine different reading behaviors (e.g., random word strings, SKread Chart; sentence strings, RADNER Reading Charts). Some reading charts (e.g., IReST) exclusively measure reading speed, while others allow for additional estimation of reading parameters such as MRS, RA and CPS. Perhaps the most widely used low vision reading test is the MNREAD Acuity Chart (Legge et al., 1989) which is a continuous-text reading test, designed to estimate reading parameters while measuring reading performance. The MNREAD test records reading-aloud speed for a series of 60-character standardized sentences (including spaces) at a range of font sizes. The test records RA, MRS, and CPS (the difference between reading acuity and critical print size is called reading acuity reserve). The MNREAD Acuity Chart was initially designed as printed text with two commercially charts available for clinical and research assessment, however, an iPad application implementation has been developed and shows similar estimates of reading acuity and critical print size, but slower maximum reading speeds compared with the original chart-based test (Calabrèse et al., 2018).

While these standardized measures simulate some aspects of naturalistic reading as they use standard text that require fixational and intra-saccadic eye movements, they are spoken reading tests. Oral reading is used to measure accuracy and reading speed easily. Early evidence suggests there are no significant intra-individual changes in eye movements between oral and silent reading, but silent reading yields faster reading speeds, even in low vision observers (Ashby et al., 2012). There is limited research on the benefits of faster speeds in silent reading, but a recent study (Inhoff and Radach, 2014) compared parafoveal processing in silent versus oral reading. Results suggest that for a given processing speed of oral pronunciation, readers are fixating and processing two to three words ahead of the word they are currently pronouncing. Silent compared to oral reading may have a greater benefit from parafoveal processing (Ashby et al., 2012). A silent reading task would simulate a more naturalistic reading environment.

While silent reading is representative of real-world reading behavior, it does not assess comprehension without explicit instruction. Observers may be able to repeat the words without comprehending the meaning of the sentence regardless of reading aloud (Crossland et al., 2008) similar to random word recognition charts such as Pepper Visual Skills for Reading (VSR) (Baldasare et al., 1986) and SKread (MacKeben et al., 2015). One silent reading task that assesses comprehension is the true/false two-alternative forced choice (2AFC) reading task (Crossland et al., 2008). The true/false task uses an automated sentence generator that creates four-word sentences that are logically either true or false. To assess comprehension simultaneously with other reading parameters, the observer makes a logical true/false judgment about the sentence, which requires that they successful read all four words. This method also eliminates the need to have the observer read the sentence aloud. The automated access to an almost unlimited corpus of text also allows for repeated testing that can be self-administered if necessary. Reading speed in the true/false task did not significantly differ with reading speed estimated with MNREAD (Crossland et al., 2008).

It is challenging to determine how eye movements and oculomotor control contribute to low vision reading difficulty compared to physical characteristics of text or spatial interference from crowding in the periphery. Physical boundaries of central scotomas are often not visible to patients with maculopathies, and therefore, it is difficult to execute inter-word saccades efficiently while reading. If patients do not use a consistent and stable PRL, it is challenging to determine whether reading difficulty is largely due to inadequate peripheral location or size of text. If the primary rehabilitation goal is to improve reading speed or to train patients to adopt a PRL, selecting a reading task that minimizes eye movements might be advantageous. A task that uses rapid serial visual presentation (RSVP) reduces the need to make intra-word saccades as it measures word identification as a function of exposure duration (Rubin and Turano, 1992, 1994). Taken together, RSVP could examine reading performance independent of eye movements.

Similar to RSVP, word/non-word identification tasks (also called lexical decision tasks) (Meyer and Schvaneveldt, 1971) examine accuracy as a function of word exposure duration. Word/non-word identification tasks require observers to make a 2AFC response whether an orthographic string is a word or a non-word (for review see, Colenbrander et al., 2011). Although, word identification is not considered a traditional reading test for some (Campbell and Butterworth, 1985), others have argued it captures significant phonological aspects of reading behavior (Lorch, 1986). Additionally, for many tasks low vision patients do not engage in sustained reading behavior and instead attempt to read one to two words at a time (e.g., prescriptions, ingredients and bills), and for these tasks, the speed of identification for one word may be more relevant than sustained narrative comprehension. We implemented a word/non-word task that uses an orthographic wordform database that includes all English word forms from a COBUILD corpus of both written and spoken text (Medler and Binder, 2005). We used the database to generate non-word letter strings syntactically matched in English. The word/non-word identification task therefore involves the recognition of a single word and the meaning, and it also allows for repeated testing and can be self-administered.

To evaluate the utility of different reading methods compared to the standardized reading test MNREAD, we estimated the reading parameters MRS, CPS, and RA in the true/false, word/non-word tasks and a computerized MNREAD test. Given the different nature of these three tasks, we understand this may be an apples and oranges comparison. However, we do not know the degree to which these tasks may be similar, whether a simple conversion factor can be used to relate the results of different tests and whether these tests may serve as an important clarification for reading outcomes in low vision rehabilitation. Additionally, two of these tasks also have the potential to be self-administered, away from the clinic, to monitor visual impairment remotely with minimal burden on the patient to schedule clinic visits. If effective, they may serve as valuable alternative endpoints in low vision rehabilitation.

## Materials and Methods

### Participants

Twelve normally sighted adults (6 female/6 male) from the student population at Northeastern University participated in this study. Participant ages ranged from 18 to 24 years, and the median age was 19 years. Participants were native English speakers with self-reported normal or corrected-to-normal habitual vision. No participants reported reading or cognitive impairment. Written informed consent was obtained from all participants prior to testing. Procedures of this study were in accordance with the Declaration of Helsinki, and approved by the Institutional Review Board at Northeastern University.

Stimuli were generated and presented with MATLAB (version R2016) and the Psychophysics Toolbox-3 (Brainard, 1997; Pelli, 1997; Kleiner et al., 2007). Stimuli were displayed on an ASUS monitor (model: VW266H; refresh rate: 60 Hz; resolution: 1920 × 1080), controlled by a Dell computer (model: Optiplex 9020). The height of the monitor was adjusted individually for each participant, so the center of the screen was at eye level. All testing was conducted binocularly in a lit room (illuminated uniformly by overhead lighting system) at a viewing distance of 88 cm. Screen luminance was set to <1cd/m^2^ min and 200 cd/m^2^ max. Letters were displayed in Times Roman font, to match the font in MNREAD, at the center of the screen. All lexical stimuli were presented as black letters against a white background with no punctuation.

### MNREAD

**FIGURE 2:**
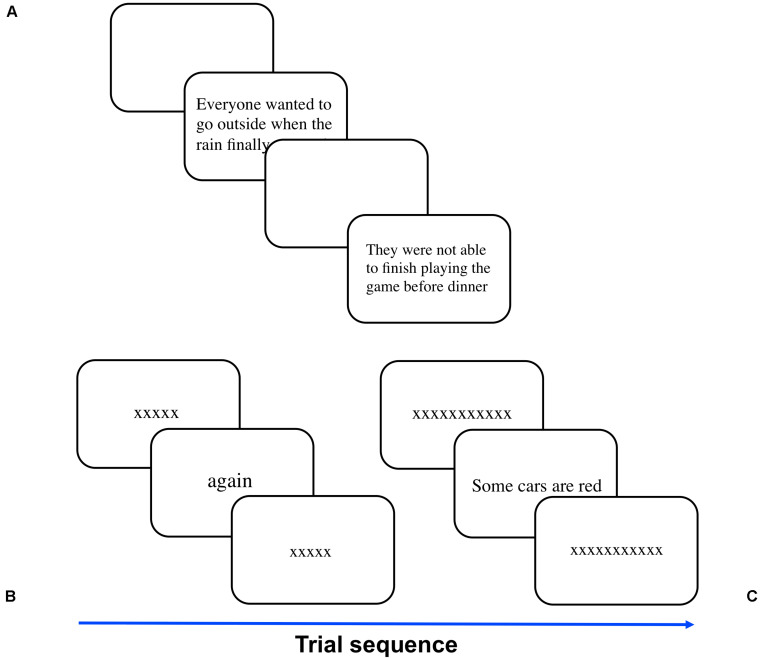
Illustration of the three reading tasks. **(A)** Observers were timed while they read aloud sentences selected from the 38 items from MNREAD charts 1 and 2. The font size decreased each time the observer successfully read a sentence until the font was too small. In 2AFC tasks, observers reported whether **(B)** pentagrams were words or non-words or **(C)** 4-word sentences were logically true or false. Font size conditions were randomly interleaved and a staircase adjusted the presentation duration for each font size condition. The pentagrams or sentences were pre- and post-masked with a string of X’s of the same size and length as the test stimuli.

MNREAD acuity charts (Mansfield et al., 1996) 1 and 2 (total of 38 sentences) were adapted for computer presentation to match the 3-line presentation format and scaled in size to logMAR (−0.5 to 1.3 in 0.1 steps) text size in point. The sentences were randomly assigned to each tested font size for each participant. Start and end time was recorded with a high-precision PsychToolbox timer [*GetSecs()*]. The experimenter initiated each sentence presentation with a keypress, and the sentence was immediately displayed. The experimenter initiated each sentence presentation with a keypress, and the sentence was immediately displayed (Figure 2A). We instructed participants to read the sentence out loud as quickly and accurately as possible. The experimenter pressed a key to end the trial after the participant completed or attempted reading the sentence. Reading time was recorded in seconds, and the experimenter documented the number of errors for each sentence. Following the MNREAD instruction manual, the test stopped when the participant could no longer attempt a sentence read due to small text size.

### True/False

Following the methods described in Crossland et al. (2008), participants indicated whether a four-word sentence was logically true or false. The sentence database was obtained from Crossland et al., and the material was screened by 3 United States nationals to convert British English terms (e.g., “teetotal”) to American English terms (e.g., “sober”) for the current participant pool. At the beginning of the task, a sequence of twenty X’s was displayed horizontally at the center of the screen along with testing instructions at the top of the screen. This sequence of X’s was purely illustrative. We instructed the participant to make a right-button mouse response if the test sentence was logically true or a left-button mouse response if the test sentence was logically false. When ready, the participant clicked either mouse button to initiate the first trial. At the start and end of each trial, the true/false sentence was pre- and post-masked with a sequence of X’s of the same font size and character length (including spaces) as the test sentence (Figure 2C). The pre-mask was presented for 0.5 s before the test sentence. The post-mask remained on the screen until the participant made a response. The participant had an unlimited amount of time to make a response. There was no initial fixation before the pre- mask.

We tested four font size conditions fixed at 0.08°, 0.11°, 0.15° or 1.63° (equivalent to −0.01, 0.11, 0.23, or 1.3 logMAR; 8, 11, 16, or 178 Pt), and randomly interleaved across trials. For each font size condition, the exposure duration of the test sentence was controlled by a 2 down 1 up rule adaptive staircase method converging at 70.7% correct (Wetherill and Levitt, 1965). The duration of the first sentence was 1, 0.8, 0.5, or 0.5 s (equivalent to 240, 300, and 480 words per minute) for font sizes 0.08°, 0.11°, 0.15°, or 1.63°, respectively). We selected these initial durations based on previous literature (Mansfield et al., 1996; Calabrèse et al., 2018). Given the expected reading function, we anticipated reading speeds to asymptote around 0.1 logMAR, and therefore we held initial durations for the two largest font sizes constant. If the participant’s response was incorrect, the duration was increased by 0.1 log units (1.2589 linear units), if their response was correct, the duration was decreased by 0.05 log units (1.1220 linear units). Exposure durations were quantized to the 16.67 milliseconds duration of each display frame. The participant then made a true/false judgment if the sentence was logically true or false with a corresponding mouse click. Each participant completed 5 practice trials with the experimenter to ensure they understood the task instructions. Participants completed 40 trials for each font size condition, for a total of 160 trials.

### Word/Non-word

The set of words and non-words were generated using the MCWord database with trigram statistics constrained to match English orthography (Medler and Binder, 2005). At the beginning of the word/non-word identification task, a sequence of five X’s was displayed horizontally at the center of the screen along with some testing instructions at the top right of the screen. Similar to the true/false task, this sequence was purely illustrative. Procedures were the same as the true/false task but instead of a sentence, a single 5-letter string word or non-word was presented at the center of the screen (Figure 2B). The participants identified whether the pentagrams were words or non-words, by clicking the right mouse button for word and left mouse button for non-word. Based on pilot testing, the initial exposure durations were 0.5, 0.25, or 0.2 s for font sizes 0.08°, 0.11°, 0.15°, or 1.63°, respectively.

### Pilot Study

We also conducted a pilot study with three experienced observers familiar with the tasks to confirm that reading performance with true/false and word/non-word tasks conformed to an exponential function. The true/false and word/non-word tasks were identical to those completed by the naïve observers with the exception that a full range of font sizes matching those used in MNREAD was tested. The font size was fixed at 0.03°, 0.05°, 0.08°, 0.11°, 0.16°, 0.19°, 0.29°, 0.38°, 0.56°, 0.66°, 0.84°, 1.03°, 1.21°, or 1.66° (equivalent to −0.50, −0.26, −0.01, 0.12, 0.27, 0.37, 0.54, 0.66, 0.90, 1.00, 1.09, 1.16, or 1.30 logMAR; 2, 4, 8, 11, 16, 20, 30, 40, 60, 70, 90, 110, 130, or 178 pt), randomly interleaved across trials.

### Data and Statistical Analysis

Data from the true/false task and the word/non-word task for each font size condition were fit with a cumulative Gaussian psychometric function (MATLAB function *fit()*: function weighted by the binomial standard deviation of responses for each exposure duration). We were concerned that observers would make mistakes even at long presentation durations for the true/false and word/non-word tasks, owing to logical errors or unfamiliarity with some words. Therefore, we included a finger slip parameter that was free to vary between 0 and 0.1. Before attempting to fit the psychometric function for each font size, we completed a binomial test to estimate if the observer performed at significantly greater than chance levels across all test durations. If performance was not significantly greater than chance for each font, the log reading speed was assigned an arbitrary value of 1.0 (10 wpm). Threshold exposure durations (secs), for word(s) at a given size were estimated at the 75% correct point on psychometric functions. We converted threshold duration estimates to words per minute. Threshold duration estimates for each font size condition in all three tests were then fit with an exponential function:

$$\log\left(R\,S\right)=A\,D+{\left(1-A\,D\right)}^{*}\,e\,x\,p^{\left(-\frac{\log\left(F\,S\right)-\text{log}\,\left(R\,A\right)}{10^{T}}\right)}$$

*RS* is reading speed, *FS* is font size, *AD* is asymptote duration, *RA* is reading acuity and *T* is the time constant of the exponential function (Campbell and Butterworth, 1985).

Exponential reading functions were fit to the data that included only the smallest font size where reading speed (log WPM) was 1.0 or greater (Cheung et al., 2008). From the reading function, we estimated reading acuity (logMAR), critical print size (logMAR) and maximum reading speed (words per minute). Reading acuity (RA) corresponds to the smallest text size that can just be read. Maximum reading speed (MRS) was calculated as the asymptotic duration of the fit, corresponding to fastest reading speed that can be achieved. A criterion of 95% of MRS was chosen to obtain the critical print size (CPS).

Cheung et al. (2008) analyzed MNREAD data (reading speed as a function print size) and compared the fits of a two-limb function and an exponential-decay function. Overall, the exponential-decay function provided slightly better fits than the two-limb function, although differences between the two fits were small. Importantly, the advantage of fitting the data with exponential-decay function is dependent on the criterion for the CPS. As a result, we selected a conservative criterion for our CPS at 95% of the MRS. The CPS estimated using two-limb function might underestimate the print size required for reading at MRS, which might influence the magnification required for optimal reading performance (Cheung et al., 2008). We compared the goodness of fit (R^2^, RMSE, and SSE) for each observer for each task, and there were no significant differences between fitting functions, *p*’s > 0.05, except the RMSE for word/non-word task. CPS estimated with two-limb function produced lower CPS estimates for both MNREAD and the true/false task, but not for the word/non-word task. Based on the overall results, we opted to fit our reading data with an exponential-decay function and a conservative criterion for CPS.

We measured the effect of task condition (MNREAD vs. True/False vs. Word/Non-word) on three estimated parameters using separate Univariate ANOVAs. Reading parameters were the dependent variables, and task conditions were fixed variables. We also measured the correlations among reading performance parameter estimates for the three tasks.

## Results

**FIGURE 3:**
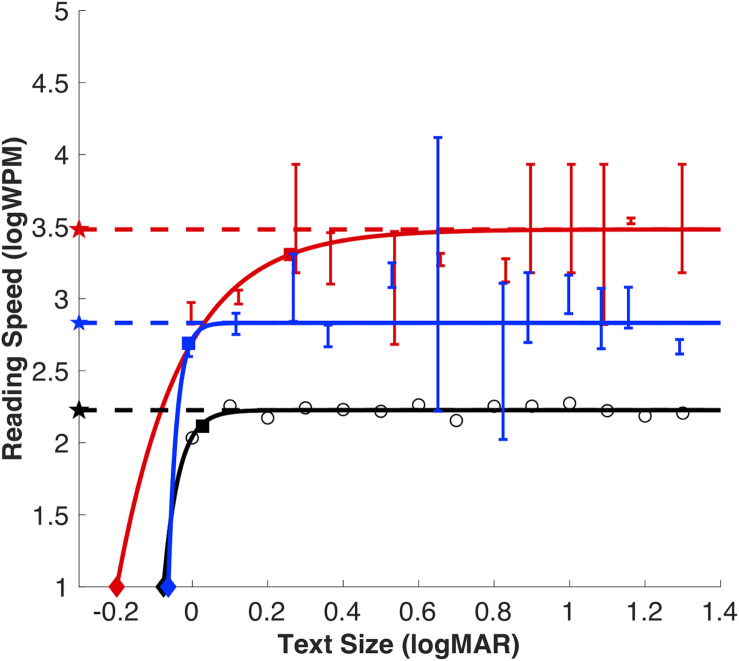
Illustration of exponential reading function from a representative for each reading task in the pilot study: True/false (blue), word/non-word (red) and MNREAD (black; raw data as open red circles). Reading estimates for MRS (dashed line with filled stars), CPS (filled squares with dashed lines) and RA (filled diamonds). Threshold duration estimates for true/false and word/non-word tasks plotted as solids lines in their respective colors, error bars show 95% confidence intervals.

**FIGURE 4:**
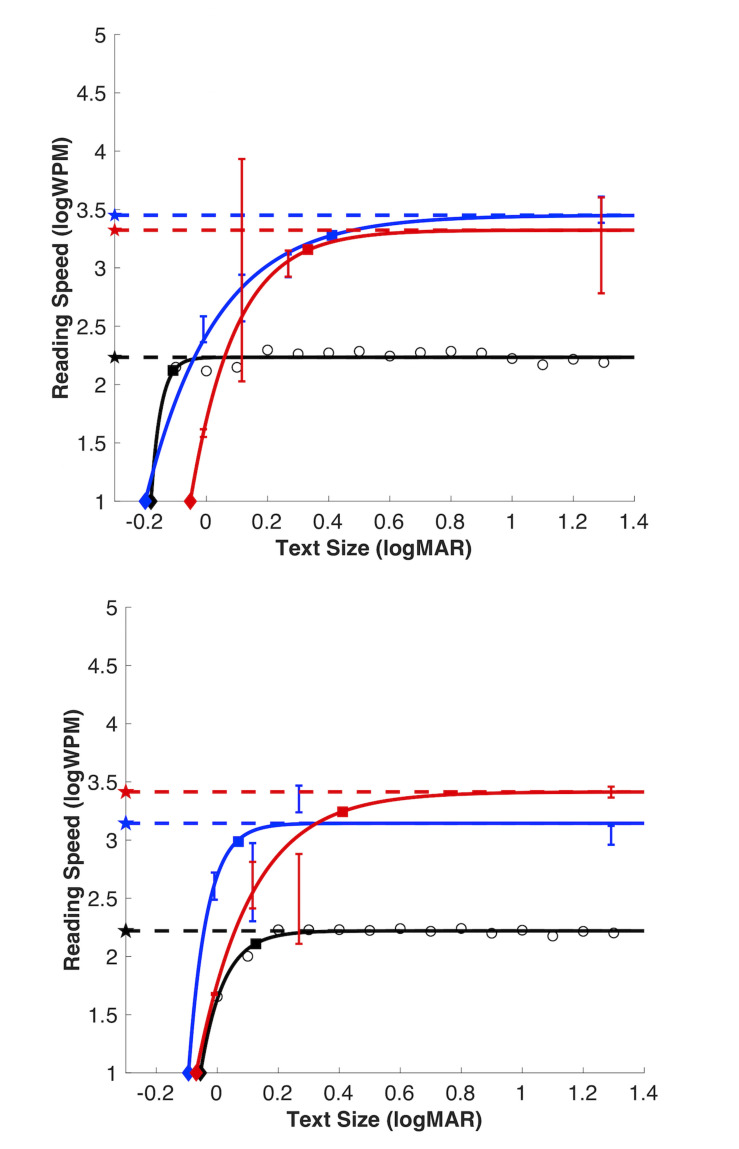
Illustration of the exponential reading functions from two naïve participants for each reading task: True/false (blue), word/non-word (red) and MNREAD (black; raw data as open red circles). Reading estimates for MRS (dashed line with filled stars), CPS (filled squares with dashed lines) and RA (filled diamonds). Threshold duration estimates for true/false and word/non-word tasks plotted as solids lines in their respective colors, error bars show 95% confidence intervals.

We removed one observer from the present analysis (*n* = 11). This observer consistently performed at 0% correct even at the largest font sizes, suggesting they switched button responses. For the true/false and word/non-word tasks, threshold stimulus durations were converted to reading speed in words per minute (note that threshold duration for the true/false task represented 4 words). Representative reading speed data for each task from the pilot study are shown for one observer (Figure 3) in black for MNREAD, blue for true/false and red for word/non-word. Performance at font sizes 2 and 4 point were not significantly better than chance, and therefore threshold estimates were not included in the exponential fit. Representative reading speed data for each task from the study with naïve observers are shown for two observers in Figure 4. In all three tasks, reading speed increased rapidly with font size above reading acuity, then saturated at a maximum reading speed, beyond which there was no further increase in reading speed. Since there was no change in performance between 0.23 and 1.3 logMAR in the pilot study, these fonts were not tested in naïve observers. This allowed for shorter testing time.

**FIGURE 5:**
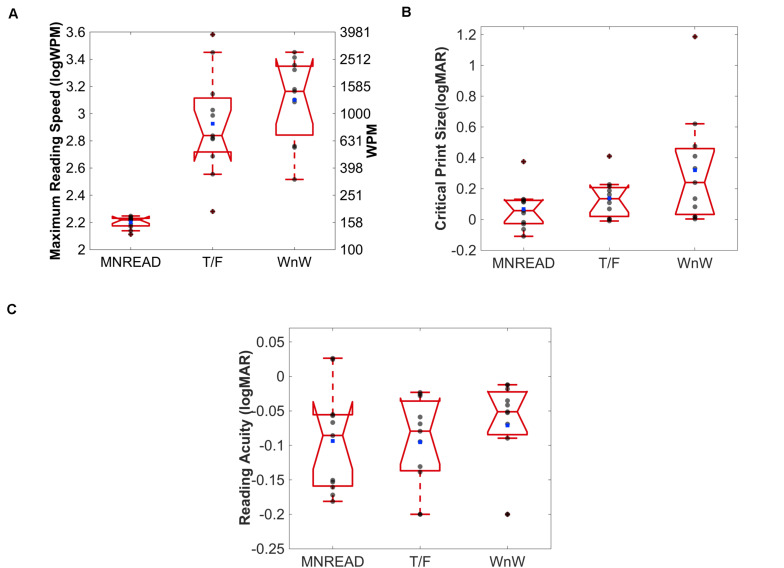
Boxplots for median maximum reading speed **(A)**, critical print size **(B)** and reading acuity **(C)** for each reading task. Solid red line represents the median, and the notch represents the 95% confidence interval of the median. The upper and lower whiskers represent values outside 50% of the data. Individual observers illustrated as gray scatter points, and blue box as the mean.

There was a significant effect of task on maximum reading speed (MRS), *F*_(__2_,_30__)_ = 31.90, *p* < 0.001). MRS was significantly faster for word/non-word (3.10 logWPM; 95% CI [2.90, 3.31]) and true/false (2.93 logWPM; 95% CI [2.67, 3.18]), compared to the MNREAD (2.20 logWPM; 95% CI [2.17, 2.23]; Bonferroni correction, *p* < 0.001). Although, reading speeds were faster for word/non-word (median = 3.16 logWPM) compared to the true/false reading task (median = 2.84 logWPM), this difference was not statistically significant (Bonferroni correction, *p* = 0.46) (Figure 5A). Note that in MNREAD, MRS is implicitly estimated at 100% correct, whereas in the present tasks, MRS was explicitly estimated at 75% correct. Therefore, the difference could be decreased with the selection of a higher% correct threshold duration for true/false and word/non-word tasks. An additional follow-up analysis revealed this relationship still held even when we increased the threshold reading speed to 95% correct, *p* < 0.0001.

There was a significant effect of task on critical print size (CPS), *F*_(__2_,_30__)_ = 3.54, *p* = 0.04. CPS estimates were significantly larger for word/non-word (0.32 logMAR; 95% CI [0.08, 0.56]) compared to MNREAD (0.07 logMAR; 95% CI [−0.02 0.16]; Bonferroni correction, *p* = 0.04), but not true/false (0.14 logMAR; 95% CI [0.05 0.22]; Bonferroni correction, *p* = 0.22) reading tasks.

Although, CPS was larger for true/false (median = 0.14) compared to MNREAD (median = 0.06), this difference was not statistically significant (Bonferroni correct, *p* = 1.0) (Figure 5B). The CPS outlier (beyond the maximum value) for word/non-word and true/false tasks is the same observer. This observer is also the MRS outlier in the true/false task and one of the more extreme scatter points on the upper whisker for word/non-word task.

There was no statistically significant effect of task on reading acuity, *F*_(__2_,_30__)_ = 0.42, *p* = 0.70. RA estimates were the largest for word/non-word (−0.07 logMAR; 95% CI [−0.12 0.03]; median = −0.02). RA estimates were similar for true/false (−0.095 logMAR; 95% CI [−0.14 −0.05]; median = −0.08) and MNREAD (−0.094 logMAR; 95% CI [−0.15 −0.04]; median = −0.09) (see Figure 5C).

Correlations between estimates of MRS, CPS and RA for each task are shown in Table 1. None of the correlations were significant. For MRS, there were positive but non-significant relationships between all three tasks. For CPS, there were positive but non-significant relationships between all three tasks. For reading acuity estimates, there were negative but non-significant relationships between all three tasks.

**TABLE 1:** Correlations between parameters estimated for each reading task. Pearson correlation coefficient and *p*-value reported for each correlation.

**Reading speed**	**MNREAD**	**True/False**	**Word/Non-word**
MNREAD	–	*r* = 0.43, *p* > 0.05	*r* = 0.36, *p* > 0.05
True/False	*r* = 0.43, *p* > 0.05	–	*r* = 0.39, *p* > 0.05
Word/Non-Word	*r* = 0.36, *p* > 0.05	*r* = 0.39, *p* > 0.05	–
**Critical print size**	MNREAD	True/False	Word/Non-word
MNREAD	–	*r* = 0.03, *p* > 0.05	*r* = 0.57, *p* > 0.05
True/False	*r* = 0.03, *p* > 0.05	–	*r* = 0.14, *p* > 0.05
Word/Non-Word	*r* = 0.57, *p* > 0.05	*r* = 0.14, *p* > 0.05	–
**Reading acuity**	MNREAD	True/False	Word/Non-word
MNREAD	–	*r* = −0.19, *p* > 0.05	*r* = −0.01, *p* > 0.05
True/False	*r* = −0.19, *p* > 0.05	–	*r* = −0.37, *p* > 0.05
Word/Non-word	*r* = −0.01, *p* > 0.05	*r* = −0.37, *p* > 0.05	–

## Discussion

Many people with low vision list reading as a primary problem and improvements in reading as a goal of rehabilitation (Elliott et al., 1997). Evidence-based methods of evaluating low vision rehabilitation interventions therefore require effective methods of reading assessment. Ideally, reading assessment tests for low vision rehabilitation should quantify key vision-related parameters of reading performance that may differ from reading tests for developmental assessment, and support repeated testing at multiple time points during intervention with automated administration. However, current methods of reading assessment fail to meet the requirement for outcome assessment in low vision rehabilitation because there is a lack of consensus on which aspects of reading should be tested (Baldasare et al., 1986; Legge et al., 1989; Rubin and Feely, 2009; Yu et al., 2010; Harvey and Walker, 2014) (for review see Rubin, 2013). Existing tests are typically chart-based with a limited corpus of text available for functional assessment and require an administrator to record reading speed. There is yet to be a standardized reading method with automated access to a near-unlimited corpus of text that automatically quantifies vision-related reading performance.

We estimated reading performance with three different reading methods, two of which have access to an almost unlimited corpus of text available for testing and can be self-administered. Similar to MNREAD, true/false and word/non-word are sensitive reading methods in which vision-related metrics of reading ability such as maximum reading speed (MRS), critical print size (CPS) and reading acuity (RA) can be estimated, but which are not confounded by memory. We found similar estimates across tasks for some but not all of these reading metrics.

In this study, we tested a sample of normally sighted young adults. MRS was significantly faster for word/non-word reading task, with reading speeds on average at 1260 wpm, and the fastest reader achieving 2818 wpm. These results are consistent with previous literature on reading speed estimates using rapid serial visual presentation (RSVP) that minimizes the need for eye movements like intra-word saccades during reading (Chung, 2002). The true/false task also achieved significantly higher reading speeds than the MNREAD with a mean MRS of 832 wpm, although, not statistically different from the word/non-word task. These results are similar to those reported by Crossland et al. (2008) who found comparable reading speeds for true/false and MNREAD when presenting each test word in isolation. Is it important to note that we adapted the printed MNREAD charts for digital presentation, and reading speeds might differ in digital vs. printed text. Our MRS for MNREAD was similar to the recently designed iPad MRS with only a difference of 0.02 logWPM (2.20 vs. 2.22 logWPM, respectively) (Calabrèse et al., 2018).

Although, maximum reading speeds were faster for word/non-word and true/false reading tasks, larger text sizes were necessary to achieve those maximum reading speeds. Critical print size is an important metric for low vision rehabilitation because it determines the smallest magnification that can be prescribed to return the greatest benefits in reading speed. On average, we estimated the CPS to be 0.23 log units larger than the RA to reach 95% of the MRS. In line with previous research, our CPS for the true/false reading task averaged 0.14 logMAR (0.12°), which is within the range (0.1–0.2°) found from previous RSVP studies (Brainard, 1997; Medler and Binder, 2005). On average, our CPS estimate (0.07 logMAR) for MNREAD was very similar to the iPad MNREAD from with only a 0.01 logMAR difference (Calabrèse et al., 2018).

Reading acuity estimates were not significantly different across all reading tasks tested here. RA was slightly lower for true/false than MNREAD and word/non-word, but only amounted to differences of 0.04 and 0.01, respectively. RA may therefore provide a general reading metric that is relatively independent of the test that is used to assess it, particularly as an outcome for reading rehabilitation across different presentation modalities or rehabilitation providers. Our results suggest that if the objective reading assessment is to determine reading acuity, then the three methods examined in this paper may be interchangeable.

There are several advantages to using an automated and unlimited text generator in addition to those previously discussed. A printed chart requires the experimenter to use a stopwatch to record the start and end of each test stimulus, which allows for potential measurement errors in timing and a reduction in test-retest repeatability. For instance, there may be a variable delay between the time the stimulus is presented to the participant and the time the timer is initiated, which may underestimate the reading time, and thus overestimate the reading speed (Xu and Bradley, 2015; Calabrèse et al., 2018). Conversely, computerized tests have accurate and precise control over stimulus timing, resulting in a more accurate measuring of reading speed. The true/false and word/non-word tasks may also be applied to other adaptive methods, such as the qReading method, a Bayesian adaptive method previously validated to measure foveal and peripheral RSVP reading performance (Hou et al., 2018; Shepard et al., 2019). One goal of the present study was to identify the relationship among performance estimates of the three tests in order to enable comparison across studies and to estimate MRS from one test based on the MRS from a different test. However, the present study showed that there was no significant correlation between estimates of MRS, CPS or RA. This finding suggests that a simple transformation may not be possible and the idiosyncratic factors affect reading performance in the different tasks.

One disadvantage of the true/false task is that the algorithm often generates peculiar and unique syllogisms in its true/false sentences. When judging the logical argument of the sentence to be true or false, observers must interpret the statements at face value, ignoring the odd semantic content of the stimuli. Despite these explicit instructions, some observers reported that they found the task cognitively challenging, and this difficulty might confound visual factors with cognitive factors of reading performance.

Given the variability of low vision conditions and individual differences within a single condition, like macular degeneration, it is challenging to predict how low vision patients would perform on these tasks based on our results from healthy observers. However, the purpose of this study was to determine the feasibility of estimating reading parameters for all three tasks. Establishing this first would help us understand if differences observed in low vision patients are due to individual differences and not the estimation of reading parameters in general. Previous evidence on the success of measuring serial word presentation (Yu et al., 2010) in the periphery, suggest the word/non-word task might be a suitable for measuring reading speed in individuals with central vision loss. Additionally, single word identification tasks allow for reading speed estimation independent on eye movements that may influence speed, such as regressive saccades (Rubin and Feely, 2009).

## Conclusion

We compared multiple metrics of reading performance with three different tasks. The results showed that while reading acuity is independent of reading task, maximum reading speed and critical print size systematically vary with reading task. These findings suggest that different reading tasks quantify distinct aspects of reading behavior and the preferred assessment method may depend on the goal of low vision rehabilitation intervention.

## Data Availability Statement

The raw data supporting the conclusions of this article will be made available by the authors, without undue reservation.

## Ethics Statement

The studies involving human participants were reviewed and approved by the Institutional Review Board of Northeastern University. The patients/participants provided their written informed consent to participate in this study.

## Author Contributions

TA contributed to the design, data collection and analyses, and manuscript writing. PB contributed to the design, data analyses, and manuscript editing. DY and Z-LL contributed to the design and manuscript editing. All authors contributed to the article and approved the submitted version.

## Conflict of Interest

The authors declare that the research was conducted in the absence of any commercial or financial relationships that could be construed as a potential conflict of interest.
